# Novel Ni-P-Tribaloy Composite Protective Coating

**DOI:** 10.3390/ma16113949

**Published:** 2023-05-25

**Authors:** Ahmed Mabrouk, Zoheir Farhat

**Affiliations:** Department of Mechanical Engineering, Dalhousie University, 1360 Barrington Street, Halifax, NS B3J 2X4, Canada

**Keywords:** Ni-P-Tribaloy composite coating, electroless Ni-P coating, toughening mechanisms, wear, hertzian-type indentation, scratch

## Abstract

Oil and gas pipelines are subject to various forms of damage and degradation during their operation. Electroless Nickel (Ni-P) coatings are widely employed as protective coatings due to their ease of application and unique properties, including high wear and corrosion resistance. However, they are not ideal for protecting pipelines due to their brittleness and low toughness. Composite coatings of higher toughness can be developed through the co-deposition of second-phase particles into the Ni-P matrix. Tribaloy (CoMoCrSi) alloy possesses excellent mechanical and tribological properties making it a potential candidate for a high-toughness composite coating. In this study, Ni-P-Tribaloy composite coating consisting of 15.7 vol.% Tribaloy was successfully deposited on low-carbon steel substrates. Both the monolithic and the composite coatings were studied to evaluate the effect of the addition of Tribaloy particles. The micro-hardness of the composite coating was measured to be 6.00 GPa, 12% greater than that of the monolithic coating. Hertzian-type indentation testing was carried out to investigate the coating’s fracture toughness and toughening mechanisms. The 15.7 vol.% Tribaloy coating exhibited remarkably less severe cracking and higher toughness. The following toughening mechanisms were observed: micro-cracking, crack bridging, crack arrest, and crack deflection. The addition of the Tribaloy particles was also estimated to quadruple the fracture toughness. Scratch testing was performed to evaluate the sliding wear resistance under a constant load and a varying number of passes. The Ni-P-Tribaloy coating exhibited more ductile behavior and higher toughness, as the dominant wear mechanism was identified as material removal, as opposed to brittle fracture in the Ni-P coating.

## 1. Introduction

The global dependence on pipelines in major applications, such as in the oil and gas industry, during production and distribution, has prompted the need for the development of new ways to protect pipelines from failure and prolong their service life [1]. Pipelines are subject to various forms of service-induced damage and degradation, including uniform and/or localized metallurgical failures [2]. Pipeline transport is a safe and economical way of transporting oil and gas compared to other means of transportation. However, pipeline failure has the potential to cause significant financial and environmental losses [3,4]. The application of electroless coatings is one protective measure adopted to minimize these losses due to their unique properties and ease of application [5].

Electroless Nickel (Ni-P), first popularized by Brenner and Riddell in 1946, is an autocatalytic plating method through a chemical reduction in an aqueous solution, with the driving force being supplied by a reducing agent, typically sodium hypophosphite [6,7,8]. Ni-P plating is widely used in several industries due to its favorable properties, including high wear and corrosion resistance, hardness, lubricity, and adhesion, allowing for the protection of parts in severe conditions [9,10]. However, Ni-P coatings are brittle and have low toughness, which limits the coating’s suitability for use as pipeline coatings where high scratch and dent resistance are required [11,12]. Recent research has proven that the co-deposition of second-phase particles into the metallic Ni-P matrix can enhance specific properties, and this leading trend in research has resulted in the development of some Ni-P composite coatings. For example, the additions of Al_2_O_3_ and SiC particles have each increased the hardness of Ni-P, while TiO_2_ particles have enhanced the corrosion resistance [13,14,15,16,17].

The toughening of brittle materials can be achieved by the addition of second-phase particles through several toughening mechanisms that involve the interactions between cracks and said particles preventing crack initiation and propagation and, in turn, increasing the fracture toughness. Those mechanisms include micro-cracking, crack bridging, crack deflection, and crack arresting [18]. Micro-cracking allows for the dissipation of the crack-driving energy by breaking up major cracks into a series of micro-cracks. Their formation close to a large crack tip reduces the stress adjacent to it [19,20]. Crack bridging involves the absorption of propagation energy by a particle as it is plastically deformed when it comes into contact with a crack. The energy lost by the crack going through the particle reduces the crack severity [21,22]. Crack deflection is another toughening mechanism where the crack changes direction upon interacting with the particles. It can occur with or without direct contact. The deviation of the crack path consumes energy, thus depleting the driving force behind crack propagation. A crack is arrested when it loses its driving force completely upon contact with a particle [23,24,25].

In this study, indentation testing was employed as an effective method to characterize the mechanical behavior of brittle coatings. It aids in evaluating a coating’s toughness and toughening mechanisms [11]. The main types of cracks that form on brittle materials during Hertzian-type indentation tests are cone cracks and radial cracks. Cone cracks, or Hertzian ring cracks, initiate on the coating surface as a ring crack just outside the area of contact, then spread downwards and outwards, forming a cone shape. Radial cracks initiate at the edge of the contact zone and propagate outwards [11,26,27,28].

Tribaloys are a family of cobalt-based alloys with the general composition of CoMoCrSi. They are characterized by outstanding wear and corrosion resistance due to their unique chemical composition. The main alloying elements, chromium, molybdenum, and silicon, each influence the properties. The first is added to improve the corrosion resistance, while the other two are added for wear resistance [29,30]. Tribaloys contain hard intermetallic Laves phases dispersed in a solid cobalt solution, and their presence is influenced by the alloy’s composition. Molybdenum and silicon are added at levels in excess of their solubility limit to induce the precipitation of the Laves phase [31,32,33]. Among the most common commercial compositions, T-800 (Co-28.5Mo-17.5Cr-3.4Si) exhibits the largest content of the Laves phase providing it with high hardness and wear resistance [34]. The Tribaloy is an attractive candidate to be applied as a protective coating for engineering equipment, and it has already been deposited by thermal spraying and laser cladding techniques, which has presented its own challenges due to characteristics of the processes, for example, the harder alloys’ tendency for brittle fracture is amplified by the substantial thermal stresses that arise from rapid cooling during laser cladding. Research has been done in an attempt to mitigate those issues [29,34,35,36]. There is no literature on the deposition of Tribaloy particles by electroless plating. However, it is an excellent candidate to be used in the development of a wear-resistant Ni-P-Tribaloy composite coating. Improved wear resistance is achieved by enhancing the hardness and toughness properties.

The objective of this study was to characterize electroless Ni-P composite coating with the addition of Tribaloy particles on low-carbon steel substrates and to investigate its indentation and scratch behavior. Hertzian indentation testing and scratch testing were conducted to evaluate the fracture toughness and sliding wear resistance of the composite coating and compared to monolithic Ni-P coating to determine the effect of the Tribaloy additions.

## 2. Materials and Methods

### 2.1. Materials

The substrates for all experiments were AISI 1018 steel discs of 16 mm diameter and 10 mm thickness. The substrate was etched and examined under confocal laser scanning microscopy (CLSM) to examine the surface microstructure of the steel.

Commercial CoCrMo powder supplied by Nanoshel UK Ltd., Congleton, UK, was used as a second-phase particle in the composite coating. It was advertised to have a composition in wt% of Co: Bal., Mo: 29%, Cr: 17%, Si: 3.5%. Inductively coupled plasma optical emission spectrometry (ICP-OES) was performed to analyze the chemical composition of the powder. Hitachi S-4700 (Hitachi High-Tech, Tokyo, Japan) scanning electron microscope (SEM) was used to examine the powder morphology of the supplied powder.

### 2.2. Coating Preparation

To prepare the coating samples, each substrate was ground up to 600 grit SiC paper and polished using 9 μm, 3 μm, and 1 μm monocrystalline diamond suspension. The substrates were then submerged in an alkaline cleaning solution at 80 ± 5 °C for 5 min. The composition of the alkaline solution is 30 g/L sodium phosphate, 50 g/L sodium hydroxide, and 30 g/L sodium carbonate. Afterwards, the substrates were rinsed with distilled water and then immersed in 20 vol% sulfuric acid for 15 s at room temperature. Following the pretreatment steps, the substrates were rinsed with distilled water and hung horizontally in the coating solution. A commercial electroless Ni-P solution containing sodium hypophosphite as the reducing agent and nickel sulfate as the source of nickel was used as plating solutions for both monolithic and composite baths. A thin layer of Ni-P was deposited as a pre-coat layer first in order to enhance the adhesion of the composite coating. Samples were kept in a 1 L Ni-P bath for 30 min and then moved to a 1 L Ni-P bath containing 0.1 g/L of Tribaloy powder for 4 h. The bath temperature was maintained at 88 ± 2 °C and the pH at 4.7 ± 0.2. Sodium hydroxide (28–30%) solution was dripped into the plating solution periodically to adjust the pH. The plating bath was stirred at 300 RPM using a magnetic stirring bar throughout the coating process to keep the particles suspended in the solution and prevent agglomeration. The Monolithic Ni-P coatings were prepared following the same procedure except for 2.5 h deposition time and stirring at 100 RPM.

Prior to conducting the tests described in this work, coating samples were polished using 600 grit SiC paper, 9 μm, and 3 μm polish to ensure a flat surface for the tests to be carried out.

### 2.3. Coating Characterization

Coating samples were sectioned using a Buehler isomet 1000 precision saw with a diamond blade at 250 RPM and a 200 g load. X-ray Diffractometry (XRD) analysis was performed on the composite coating surface as well as the substrate, powder, and monolithic coating. Each sample was scanned with Bruker D8 Advance diffractometer (Bruker Corporation, Billerica, MA, USA) using Cu Kα radiation with a wavelength of 1.54 λ. The scan angle ranged from 20–120° to ensure that all peaks were included. Keyence (Keyence Corporation, Osaka, Japan) confocal laser scanning microscopy (CLSM), and Hitachi S-4700 Scanning Electron Microscope (SEM), equipped with Energy Dispersive Spectroscopy (EDS), was utilized to examine the surface and the cross-section of the coatings and to determine the coating composition.

### 2.4. Micro-Hardness

The hardness of each coating was measured using NANOVEA PB 1000 mechanical tester (NANOVEA Inc., Irvine, CA, USA). Micro-hardness tests were performed on the coatings’ surfaces with a Vickers indenter under an applied load of 6 N. Multiple tests were performed on each sample to ensure repeatability. Load–depth plots were generated, and the hardness (GPa), elastic modulus (GPa), and maximum indent depth (μm) were determined and compared.

### 2.5. Indentation Testing

PASCO ME-8236 (PASCO scientific, Roseville, CA, USA) materials testing apparatus was used to perform the indentation tests on the coatings’ surfaces in order to investigate their indentation behavior and crack formation. A spherical WC-6Co indenter with a radius of 0.795 mm was used to conduct the tests. A load of 2000 N was applied at an average loading rate of 0.5 mm/min. An acoustic emission (AE) sensor was attached to the samples during the tests to monitor cracking events. After indentation, samples were sectioned, and both the surface and the cross-section were analyzed using CLSM and SEM to examine the indents. The PASCO Capstone (v1.4.1) software automatically recorded the load–depth data throughout the indentation process.

### 2.6. Scratch Testing

To evaluate the wear resistance of the coating, scratch tests were carried out using Universal Micro Tribometer (UMT). A sharp diamond indenter having a radius of 0.2 mm was used to perform the scratch tests with multiple passes under a constant load of 1 kg. Each sample was subjected to five scratches with varying numbers of passes: 1, 25, 50, 75, and 100 passes. Each scratch had a sliding distance of 5 mm, and each pass took 30 s with the indenter sliding at a speed of 0.17 mm/s. An acoustic emissions (AE) sensor was attached to the indenter to monitor the acoustic signals due to cracking during scratches. CLSM and SEM were utilized to characterize the wear tracks and to calculate the volume loss associated with each sliding distance.

## 3. Results and Discussion

### 3.1. Materials Analysis

**Figure 1:**
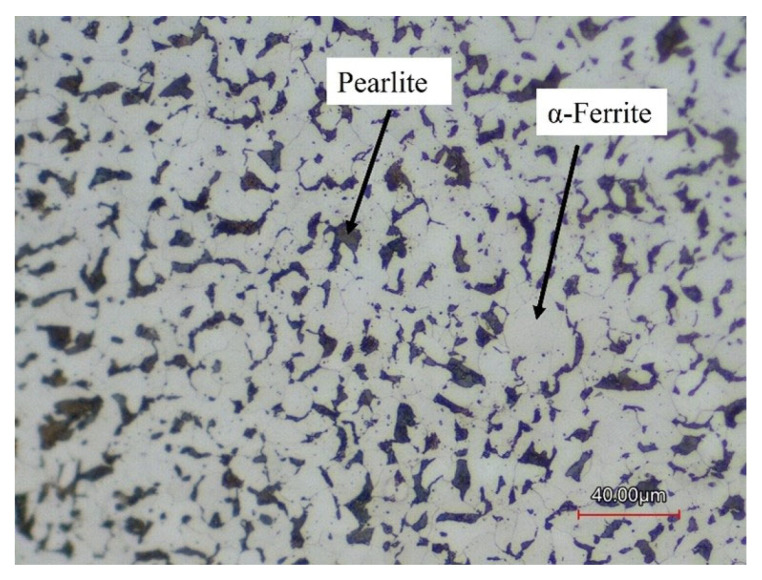
Microstructure of AISI 1018 steel.

Table 1 shows the composition of the AISI 1018 steel used as substrates. The surface microstructure of the AISI 1018 steel is shown in Figure 1 from confocal microscopy after etching of the surface. The substrate’s microstructure consists of α-ferrite and pearlite.

**Figure 2:**
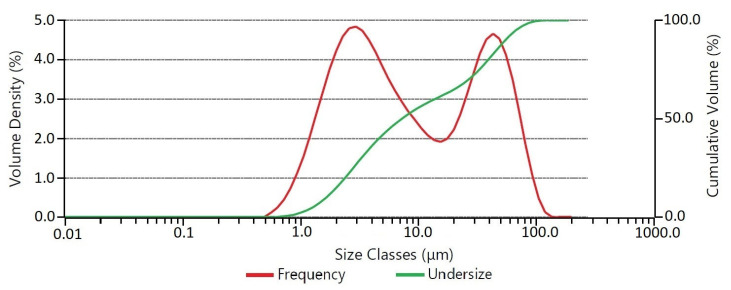
Particle size distribution.

**Figure 3:**
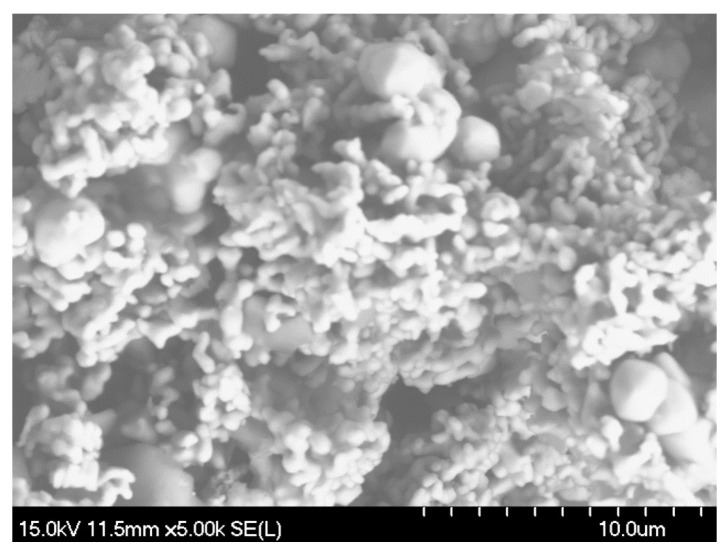
SEM image of the CoCrMo powders.

Inductively coupled plasma optical emission spectrometry (ICP-OES) was performed on the as-received CoCrMo powder. The composition in wt% was found to be Co: 50.53%, Mo: 29.15%, Cr: 17.82%, and Si: 2.50%. The particle size distribution of the powder is shown in Figure 2. The powder exhibits a bimodal particle size distribution. The two major particle sizes observed are approximately 3 μm and 40 μm, while some particles are larger than 100 μm. The values of D10, D50, D80, and D90 were found to be 1.65 μm, 7.21 μm, 39.1 μm, and 54.8 μm, respectively. The powder morphology of the supplied powder is shown in Figure 3 using SEM.

### 3.2. Coating Characterization

**Figure 4:**
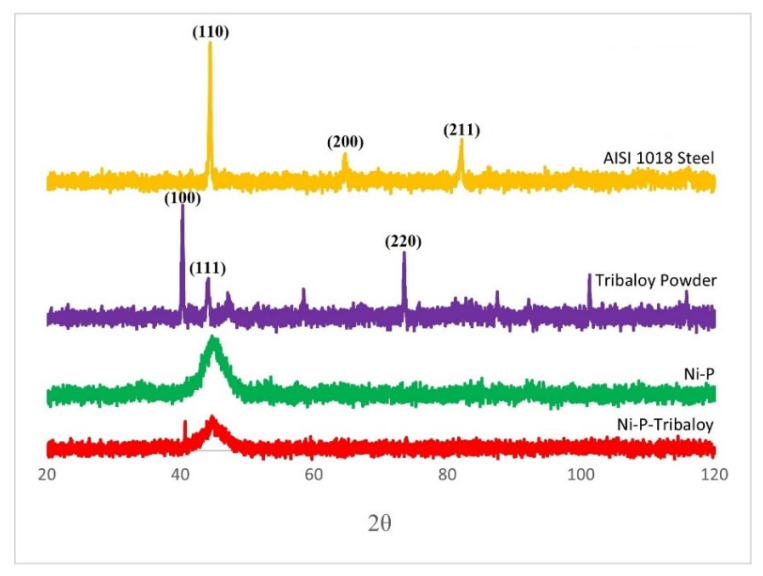
X-ray diffraction patterns of substrate, Tribaloy powder, Ni-P, and Ni-P-Tribaloy.

Composite Ni-P-Tribaloy coatings were plated on AISI 1018 steel substrates. Figure 4 shows the X-ray diffraction patterns generated from the steel substrate, Tribaloy powder, monolithic Ni-P coating, and composite Ni-P-Tribaloy coating as deposited. AISI 1018 steel reveals diffractions from the 110 and 200 planes. Here, the generated diffraction pattern shows the first two peaks occurring at 44.459° and 64.701°, which matches the Fe powder diffraction file (PDF ID: 00-006-0696). The monolithic Ni-P coating was mostly amorphous, having a broad peak at 44.775° extending from 42° to 48°, consistent with the literature and closely matching a nickel phosphide powder diffraction file (PDF ID: 04-003-6331). The broad peak in the form of a hill is a feature of amorphous materials [37]. The first high-intensity peak observed in the as-received Tribaloy diffraction pattern occurs at 40.340°, followed by a smaller peak at 44.203°. This fairly matches a Co1.539Cr0.549Mo0.912 powder diffraction file (PDF ID: 00-026-0425). The Ni-P-Tribaloy coating was found to be mostly amorphous. It exhibited a broad peak similar to that of the Ni-P diffraction pattern from 42° to 48° that includes a smaller peak corresponding to the largest peak observed in the Tribaloy powder’s diffraction pattern.

**Figure 5:**
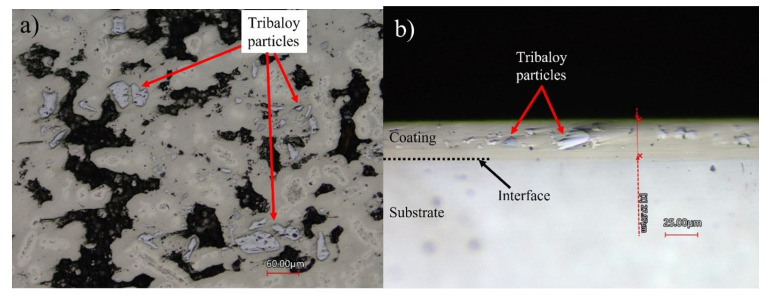
(**a**) Polished surface and (**b**) cross-section of Ni-P-Tribaloy.

**Figure 6:**
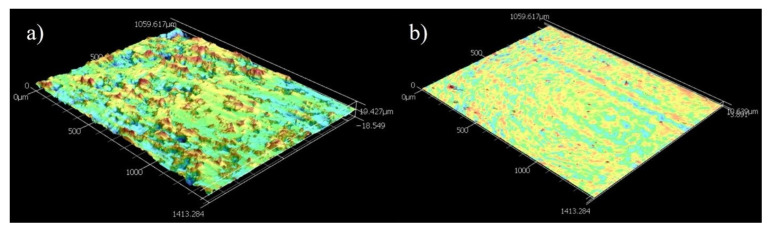
Surface topography of (**a**) Ni-P-Tribaloy and (**b**) Ni-P (at 250% height magnification).

Figure 5a,b shows images of the Ni-P-Tribaloy coating surface and cross-section after polishing. It is clear that the second-phase particles were successfully embedded in the coating matrix. The black areas seen in Figure 5a are due to the surface roughness. The coating thickness was approximately 30 μm, and the substrate–coating interface exhibited excellent bonding, as seen in Figure 5b. The addition of the Tribaloy particles resulted in a rougher surface compared to the monolithic Ni-P surface. Using the Keyence CLSM’s 3D topographic scanning, the surface roughness of the coating was measured, and the surface topography shown in Figure 6a was generated. The surface roughness Sa value was measured to be 2.30 ± 0.17 μm based on four different aerial measurements of 1.49 mm^2^. While the Ni-P coating’s Sa value was measured to be 0.430 μm, and its surface topography is shown in Figure 6b for comparison.

**Figure 7:**
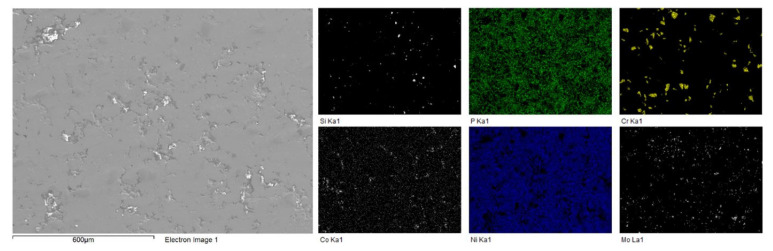
Surface EDS mapping of Ni-P-Tribaloy.

**Figure 8:**
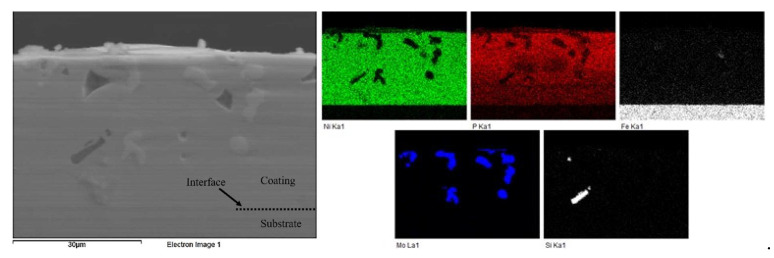
Cross-sectional EDS mapping of Ni-P-Tribaloy.

EDS analysis was performed on the surface of the composite coating. Table 2 contains the chemical composition of the coating from EDS, and Figure 7 shows the results of the elemental mapping to visualize the distribution of the elements present. This reinforces the fact that the composite coating consists of the Ni-P matrix with second-phase particles dispersed within the matrix. The presence of the four constituents of the Tribaloy was detected in the coating: cobalt, molybdenum, chromium, and silicon. The reason for the low cobalt content detected by EDS is due to peak overlap with nickel in the EDS spectra [38]. Adjusting for this based on the cobalt wt.% in the powder’s composition (Section 2.1), the cobalt content can be estimated to be 8.33 wt.% in the coating and 74.6 wt.% for the nickel. Thus, the coating can be described as having 16.5 wt.% Tribaloy, which is equivalent to 15.7 vol.%. Figure 8 shows an example of the cross-sectional EDS analysis over a smaller area. It can be seen that the particles are distinct from the matrix proving their successful incorporation in the composite coating. The substrate–coating interface is clearly defined in Figure 8 as represented by the noticeable interface between the Fe and the Ni-P matrix in the elemental map.

### 3.3. Micro-Hardness

**Figure 9:**
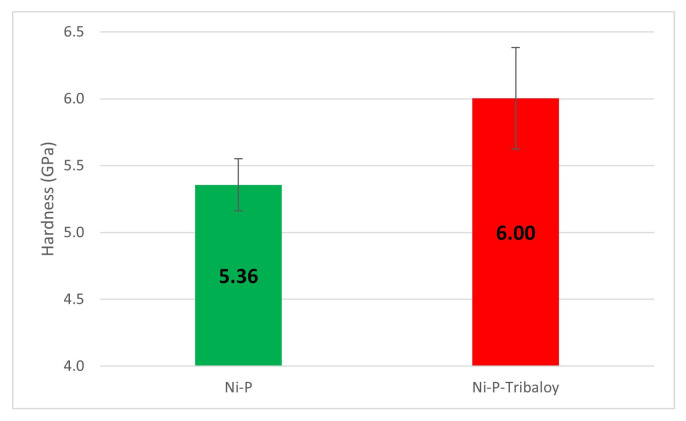
Micro-hardness values of Ni-P and Ni-P-Tribaloy.

Four micro-hardness measurements were taken on each coating sample. The average values are shown in Figure 9, with the error bars representing the standard deviation of the measurements. The monolithic Ni-P coating has a Vickers micro-hardness of 5.36 GPa, which falls within the range of the reported values of 5–6.5 GPa [39], and an elastic modulus of 125.1 GPa. The composite Ni-P-Tribaloy coating has a Vickers micro-hardness of 6.00 GPa and an elastic modulus of 135.9 GPa, exhibiting a 12% increase in hardness over the monolithic coating. The increase in hardness is attributed to the addition of the harder Tribaloy particles to the Ni-P coating. AISI 1018 steel typically has a hardness of 1.7 GPa and an elastic modulus of 205 GPa [18,40]. Both coatings increase the hardness significantly when coated over AISI 1018 steel substrates.

**Figure 10:**
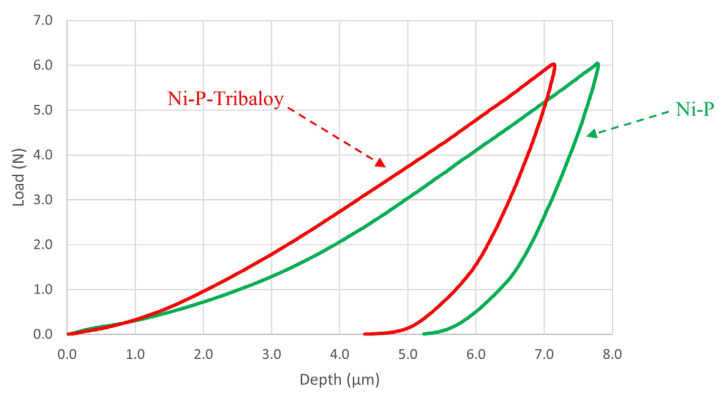
Examples of micro-indentation of Ni-P and Ni-P-Tribaloy.

On average, the maximum depth reached by the indenter was 7.73 μm in the monolithic coating samples and 7.35 μm in the composite coating samples. The indenter penetrated deeper into the monolithic coating as expected due to the higher hardness of the Ni-P-Tribaloy composite coating. Figure 10 contains representative examples of the load–depth curves produced for both types of coatings showing the difference in indent depth under a 6 N load.

### 3.4. Hertzian-Type Indentation Resistance

**Figure 11:**
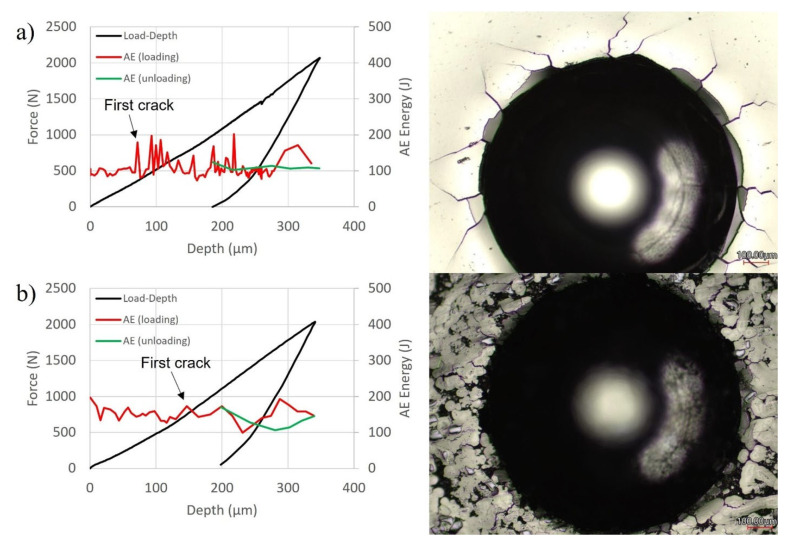
Load-depth curves with AE & confocal microscopy surface indent for (**a**) Ni-P and (**b**) Ni-P-Tribaloy.

Each coated sample was subjected to Hertzian-type indentation at a maximum applied load of 2000 N. The load–depth curves generated from both indents are shown in Figure 11, as well as microscopic images of the indents showing the crack types. Acoustic emissions energy is related to fracture energy and is established as a parameter for investigating crack initiation and propagation [41]. Therefore, the acoustic emission signals, during loading and unloading, were also collected and plotted over their corresponding load–depth curves to support the findings from microscopy. The monolithic Ni-P coating had a slightly higher penetration depth of 348 μm compared to 341 μm for the composite Ni-P-Tribaloy coating.

The area surrounding the indent on the monolithic coating shows evidence of coating delamination and large radial cracks, which are typical for the brittle Ni-P coatings [11], while the composite coating exhibited less cracking and delamination. The reduction in crack size is evidence of toughening [42]. Based on the measured AE signals, the Ni-P coating had significantly higher acoustic activity, including a large initial spike at 361 N. This confirms that it exhibited a higher severity of cracking events than the composite coating. On the other hand, there are no clear spikes in AE energy observed during the indentation of the Ni-P-Tribaloy coating. Slight changes in the AE signals can be attributed to delamination and/or the formation of small radial cracks [25]. There were no cracks detected during unloading. The area under the load-depth curve up to the first crack force of the indentation test can be used to determine the toughness of the coating [28]. The toughness of the Ni-P coating is calculated to be 13.05 mJ, corresponding to the first major crack that occurs at 361 N. For the Ni-P-Tribaloy coating, it can be assumed that the small peak at 748 N is the first significant crack. Thus, the toughness of the composite coating is estimated to be 51.80 mJ, four times higher than that of the monolithic coating.

**Figure 12:**
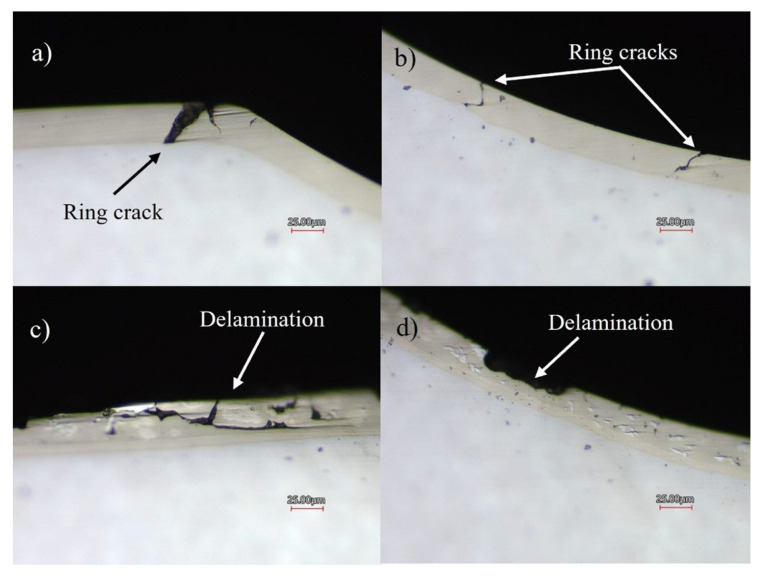
Select confocal images of indent cross-section for (**a**,**b**) Ni-P and (**c**,**d**) Ni-P-Tribaloy.

Confocal microscopy images of the indentations’ cross sections are shown in Figure 12. Figure 12a,b show severe ring cracking, typical under Hertzian-type contact [26], just outside the area of contact and inside the indent that extends across the entire layer of the Ni-P coating. Figure 12c shows delamination that developed near the edge of the indent on the Ni-P-Tribaloy, where cracks join under the surface, causing a piece of the coating to detach. Figure 12d shows another example of coating delamination with no major cracks visible, whereas the monolithic coating had severe cracks at a similar location. This is representative of the rest of the indent. Cracks observed on the cross-section of the composite coating after indentation were shallow cracks, as opposed to the monolithic coating, where cracks reached the substrate. It should also be noted that no coating delamination was observed at the substrate–coating interface.

**Figure 13:**
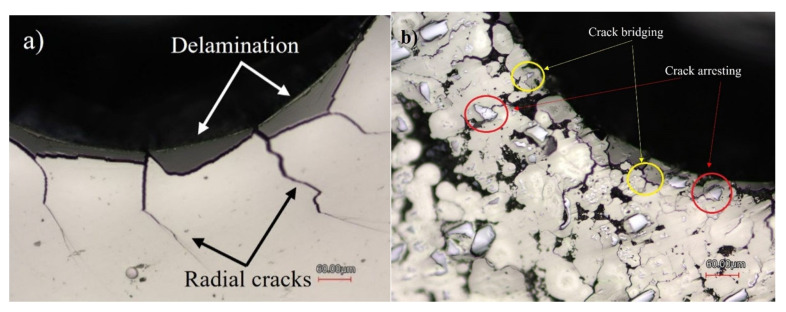
Toughening mechanisms & closeup surface indent for (**a**) Ni-P and (**b**) Ni-P-Tribaloy.

Several toughening mechanisms were found to be operating within the Ni-P-Tribaloy coating. The dominant crack mode around the indent in the Ni-P coatings were the severe radial cracks seen in Figure 13a, accompanying delamination and ring cracks. At the same time, the Ni-P-Tribaloy coating appears to have a high density of micro-cracking around the edge of the indent, shown in Figure 13b, which is supported by the AE data indicating the presence of micro-cracks over the major crack. Generally, the presence of cracks is undesirable. However, micro-cracking can reduce the initial crack propagation energy (driving force) for major cracks to grow, leading to improved toughness [42]. Figure 13b also shows instances of crack bridging and crack arrest. Upon the propagation of major radial cracks, they encountered several particles that absorbed the energy, eventually leading to limiting the severity of the cracks and, in some cases, stopping them. For a crack to pass through a reinforcement particle, energy is dissipated, thus reducing the intensity of the crack [25].

**Figure 14:**
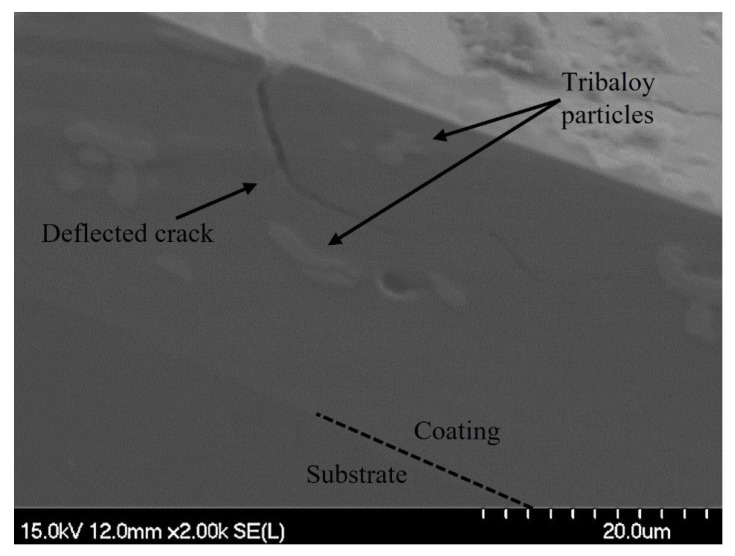
SEM image of crack deflection in Ni-P-Tribaloy.

Further evidence of toughening was found using SEM imaging. Figure 14 shows an example of crack deflection on the cross-section of the indent as a crack interacts with the second-phase particles. When a crack tip approaches a particle, it deflects and deviates in another direction, releasing energy in the process. In this case, deflection occurs without direct contact due to the compressive area forming around the particle, impeding the tensile forces associated with crack propagation [25]. The change in the crack path requires energy, and dissipating the energy available, in turn, contributes to toughening.

### 3.5. Scratch Resistance

**Figure 15:**
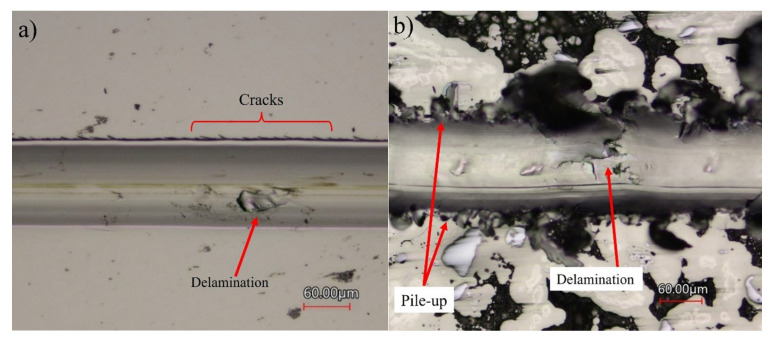
Examples of multi-pass wear tracks from (**a**) Ni-P and (**b**) Ni-P-Tribaloy.

Each coating was subjected to five scratches of varying numbers of passes. The wear tracks can be described as follows: each wear track is 5 mm long with mostly constant width throughout. A higher number of passes represents a greater degree of wear, thus wider and deeper tracks. Examples of multi-pass wear tracks from both coatings are shown in Figure 15. Visible cracks were observed on the Ni-P coating outside the wear track throughout the length of the scratch, as well as some instances of delamination inside the track. The Ni-P-Tribaloy coating showed material pile-up along the wear track and noticeably more delamination. Ni-P is known to be brittle and susceptible to cracking [43], and the Ni-P-Tribaloy coating appears to exhibit more ductile behavior under the same test conditions.

**Figure 16:**
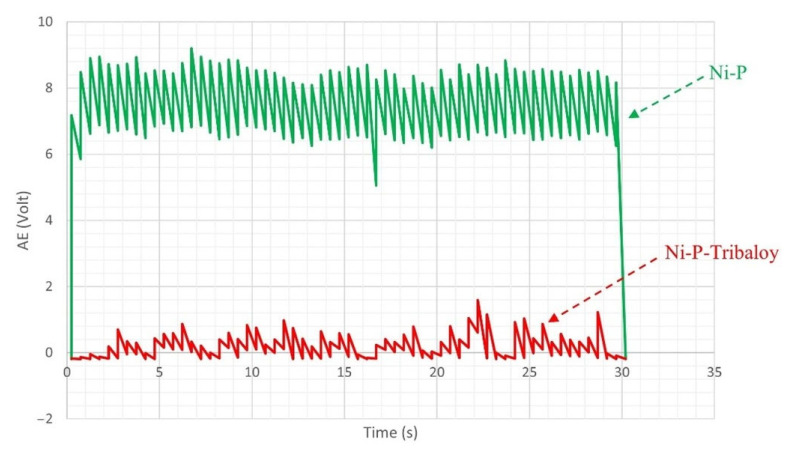
AE signals of single-pass scratch from Ni-P and Ni-P-Tribaloy.

Acoustic emissions (AE) were measured during scratching to detect signals due to cracking activity. The AE signals, in volts, during a single-pass scratch test are shown in Figure 16. It is clear that the AE signals emitted as the Ni-P coating is scratched have greater intensity and reach higher levels than that of the Ni-P-Tribaloy coating, which is free of high noise signals. This can be attributed to the formation of cracks [42]. On the other hand, the low noise signals that are detected in the Ni-P-Tribaloy sample are likely due to both micro-cracking and the surface topography, as the indenter is sliding over a particle producing those signals [42]. The Ni-P AE signals have reached up to 8.9 V in the first scratch pass, while the highest peak reached by Ni-P-Tribaloy is only 1.6 V.

**Figure 17:**
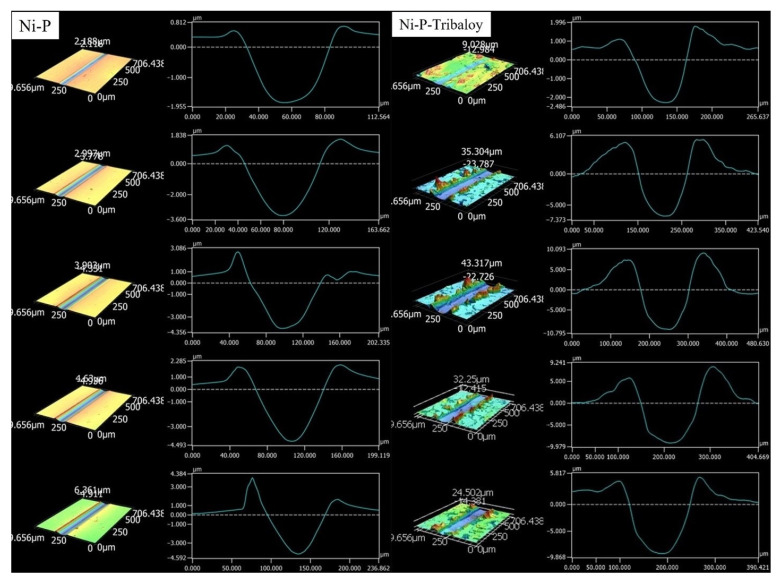
Shape and average profile of Ni-P and Ni-P-Tribaloy for 1, 25, 50, 75, and 100 passes.

Keyence CLSM’s 3D scanning was employed to analyze the wear tracks. Figure 17 illustrates the shape of the wear track for both types of coatings as the number of passes progresses. Scans of different areas revealed that Ni-P-Tribaloy coatings experience significant pile-up along the wear tracks, compared to minimal pile-up in the Ni-P coating at the same height magnification. This indicates that the composite coating exhibits higher ductility. The average profile of each scratch was generated by taking an average of 1000 lines across the scratch at 5 μm intervals, encompassing the entire 5 mm length of each scratch. The extent of material loss from the wear tracks was quantified based on their average profiles. The depths of the scratches were found to be increasing from 2.2 to 4.8 μm and from 2.9 to 9.3 μm for the Ni-P and Ni-P-Tribaloy, respectively, with an increase in the number of passes. In addition, the widths increased from 56.2 to 81.1 μm and from 84.4 to 127.0 μm, respectively.

**Figure 18:**
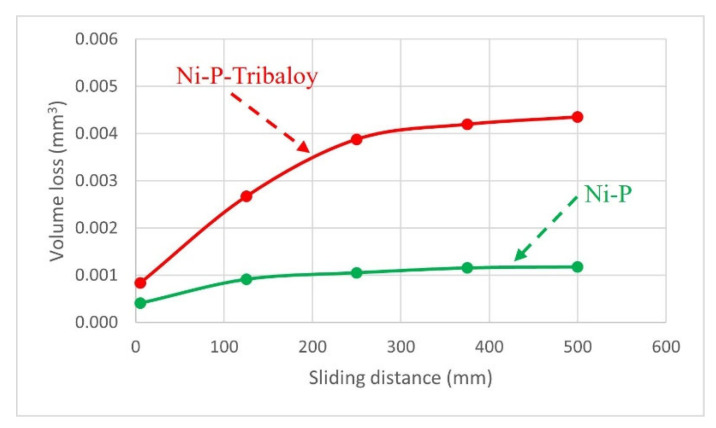
Volume loss per sliding distance for Ni-P and Ni-P-Tribaloy.

The volume loss after eliminating the pile-up effect at the edges, is plotted in Figure 18 against the sliding distance. The sliding distance is deemed as the number of passes multiplied by the length of one pass and is proportional to the amount of wear that a sample undergoes. As expected, the volume loss increases as wear progresses in both cases. It was found that the composite Ni-P-Tribaloy coating had greater volume loss than the monolithic Ni-P at each sliding distance under the same test conditions. It should be noted that the wear rate, represented by the slope of the volume loss against the scratch distance curve [43], decreases as wear progresses. The volume loss rate after 100 passes is 1.18 × 10^3^ and 4.35 × 10^3^ mm^3^/mm for the monolithic and composite coatings, respectively. The greater volume loss in the Ni-P-Tribaloy coating can be attributed to the Tribaloy particles becoming detached, leaving gaps in the coating, and more importantly, getting trapped between the indenter and the surface, causing three-body wear, which contributes significantly to the wear rate as the hard particles are wearing out the coating surface.

**Figure 19:**
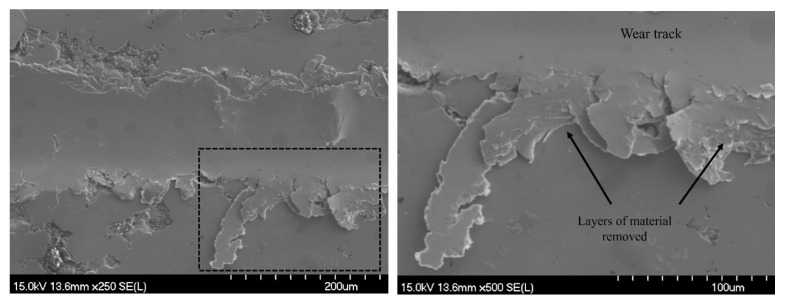
SEM image and closeup of material deformation wear mechanism for Ni-P-Tribaloy at 100 passes.

While the greater volume loss is unfavorable for the composite coating, it is important to consider the dominant wear mechanisms present in order to evaluate the wear resistance, especially when comparing a brittle material to a more ductile one, as volume loss does not account for fracture which is established to be prominent in the brittle Ni-P coatings [39]. SEM images in Figure 19 depict the wear mechanism present in the Ni-P-Tribaloy after 100 passes. It can be seen material was removed from the wear track and pushed to the sides of the track due to the plowing effect. Layers of materials were squeezed out successively with every pass by the indenter to accommodate the wear. The build-up of layers of material is pointed out in Figure 19. The dominant wear mechanism for the Ni-P coating is a brittle fracture, which does not particularly contribute to the removal of material in the same way. Instead, the energy of the scratch indenter is converted to crack initiation and propagation [25]. The abundance of pile-up along the edges of the Ni-P-Tribaloy wear tracks and the dominant wear mechanism being material removal is indicative of improved toughness and ductility.

**Figure 20:**
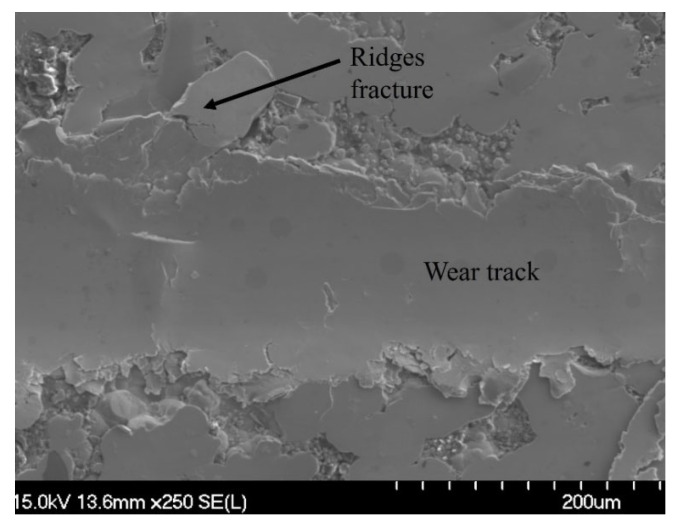
SEM image of fracture at the wear track ridges for Ni-P-Tribaloy at 100 passes.

**Figure 21:**
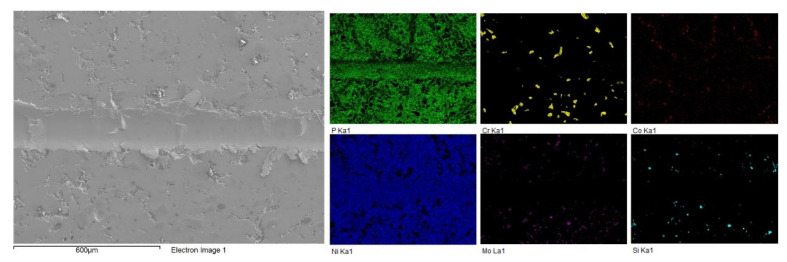
EDS mapping of the wear track for Ni-P-Tribaloy at 100 passes.

To illustrate how wear debris forms, Figure 20 shows another example of the wear track after 100 passes on the Ni-P-Tribaloy coating surface. It can be seen that material from the wear track is extruded out, forming plate-like debris with repeated sliding. Those plates then fracture and break away as debris. Further examination of the wear track by EDS is shown in Figure 21 for the 100 passes, which is the highest degree of wear in this study. It is clear from the distribution of the Tribaloy particles that they exist in considerably fewer amounts inside the wear track after the 100-pass scratch test. This is due to the particles being removed from the track as the surface is scratched repeatedly. Although the particles can be seen on the rest of the surface, they were removed from the track, while the Ni and P elements are present throughout the captured area in the figure.

## 4. Conclusions

In conclusion, electroless Ni-P-Tribaloy composite coatings were successfully deposited on low-carbon steel substrates. XRD and SEM/EDS verified the deposition of the coating and showed the distribution of the second-phase particles in the matrix. A coating thickness of 30 μm with excellent bonding to the substrate was demonstrated. The composite coating had a micro-hardness of 6.00 GPa, a 12% increase over the monolithic coating.

The indentation behavior of the 15.7 vol.% Tribaloy composite coating was found to be superior to the Ni-P coating. It exhibited remarkably less severe cracking events from acoustic emissions, a reduction in crack size on the surface, and minimal cracking along with shallow cracks on the cross-section. Ring cracks, radial cracks, and delamination were observed. The toughening mechanisms micro-cracking, crack bridging, crack arrest, and crack deflection were identified. The toughness of the composite coating was estimated to be four times higher.

Despite greater volume loss during scratching, the Ni-P-Tribaloy displayed more ductile behavior having significantly more pile-up along the edges of the tracks at each number of passes. The dominant wear mechanism was recognized as material removal and minimal cracking was observed compared to the Ni-P coating, pointing to an increase in toughness due to the addition of Tribaloy particles. The composite coating experienced greater volume loss due to the particles being removed from the wear track and causing three-body wear.

## Figures and Tables

**Table 1:** Composition of AISI 1018 Steel.

Element	C	Mn	Cu	Cr	Si	P	Fe
Weight %	0.182	0.754	0.186	0.181	0.095	0.04	Bal.

**Table 2:** Surface EDS analysis results wt.% of Ni-P-Tribaloy.

Element	Ni	P	Cr	Mo	Si	Co
Weight %	82.40	8.88	3.94	2.81	1.42	0.55

## Data Availability

Not applicable.
